# The impact of college students’ physical exercise on subjective well-being: a chain-mediation model involving psychological resilience and social support with gender as the moderator

**DOI:** 10.1186/s40359-026-04472-y

**Published:** 2026-04-01

**Authors:** Jing Shen, Yundong Xu

**Affiliations:** 1 Jiangsu Open University (The City Vocational College of Jiangsu), 832 Yingtian Street, Nanjing, Jiangsu Province 210000 China; 2 National Science Library (Chengdu), Chinese Academy of Sciences, Chengdu, 610299 China; 3 Department of Information Resource Management, School of Economics and Management, University of Chinese Academy of Sciences, Beijing, 100049 China

**Keywords:** College students, Physical exercise, Subjective well-being, Psychological resilience, Social support, Gender moderation

## Abstract

Based on the background of Healthy China, this study took 494 college students as the subjects, collected data using the Physical Activity Rating Scale, Perceived Social Support Scale, Adolescent Resilience Scale, and Subjective Well-being Scale, and constructed a chained mediation model using SmartPLS software to explore the mechanism of physical exercise on subjective well-being and the moderating effect of gender. The study found that although physical exercise cannot directly predict subjective well-being, it indirectly enhances well-being through the chained mediation path of “social support —> resilience”. This mediating effect becomes significant only when an individual’s social support translates into psychological resilience.Specifically, physical exercise can significantly enhance social support and resilience, and high levels of social support significantly positively predict resilience, which in turn directly and significantly promotes subjective well-being; Bootstrap tests confirmed the significant chained path effect of “physical exercise —> social support —> resilience —> subjective well-being”. The study also revealed that gender plays a significant moderating role in the path of “social support —> subjective well-being”: social support positively predicts subjective well-being in males but negatively predicts it in females; further moderated chained mediation tests indicated that the above chained mediation effect is only significant in the female group (effect size = 0.129, 95% CI [0.011, 0.242]), but not in the male group. The conclusion suggests that physical exercise indirectly enhances college students’ subjective well-being by enhancing social support and resilience, and this mechanism exhibits significant gender differences. It is recommended that in practice, social support networks in physical activities should be strengthened for males, while for females, attention should be paid to stress management in a high-support environment and the cultivation of their positive coping abilities. At the same time, resilience cultivation should be integrated into the physical exercise process to achieve targeted mental health promotion.

**Supplementary Information:**

The online version contains supplementary material available at 10.1186/s40359-026-04472-y.

## Introduction

Under the influence of the “Healthy China” strategy, physical activities have gained increasing attention. According to the Statistical Bulletin on National Economic and Social Development of the People’s Republic of China 2023 released by the National Bureau of Statistics, by the end of 2023, the total area of sports facilities nationwide reached 4.07 billion square meters, with the total number of facilities reaching 4.593 million. The average per capita area reached 2.89 square meters. The concept of national fitness has gained widespread acceptance alongside these rising figures. As the saying goes, “A strong nation thrives on a strong sports foundation, and the prosperity of the nation drives the advancement of sports.” Building a sports powerhouse is a crucial foundation for achieving the Healthy China goal.

At the 2020 Symposium of Experts and Representatives in Education, Culture, Health, and Sports, General Secretary Xi Jinping explicitly stated the need to accelerate the development of a sports powerhouse and tangibly enhance the people’s sense of fulfillment, happiness, and security [1]. Concurrently, the 2023 Special Action Plan for Comprehensively Strengthening and Improving Student Mental Health Work in the New Era (2023–2025), jointly issued by the Ministry of Education and 16 other departments, emphasized placing mental health work in a more prominent position. Currently, universities are promoting student mental health education through diverse approaches, with the “Five-Education Nurturing the Mind” initiative emerging as a key pathway to enhance psychological well-being. “Strengthening the Mind Through Physical Education” is increasingly playing an indispensable role in promoting student mental health. Physical activities effectively regulate emotions and alleviate psychological stress, thereby playing a positive and proactive role in students’ comprehensive development [2]. Subjective well-being is a key research area in psychology. In recent years, an increasing number of college students have exhibited behaviors such as “lying flat,” “slacking off,” and experiencing “hollow heart syndrome,” indicating concerning psychological states. For university students, subjective well-being warrants heightened attention; hence, its selection as a core focus of this study [3].

Social support represents interpersonal connections that provide individuals with abundant social resources [4]. Research indicates that individuals with higher-quality relationships and larger personal social networks experience greater well-being [5, 6]. Psychological resilience, meanwhile, refers to an individual’s adaptive capacity in facing stress and challenges, enabling emotional stability and coping with adversity. College years represent a critical stage of physical and psychological development. Subjective well-being, as a key indicator of mental health and quality of life, is gaining increasing attention. Sports communities foster positive, mutually supportive environments. Integrating into such groups allows individuals to experience an optimistic atmosphere, thereby enhancing subjective well-being. Shang Y’s research indicates that physical exercise can improve peer relationships, elevate levels of self-actualization, and ultimately lead to greater subjective well-being [7].

## Research theories and hypotheses

### Relationship between physical exercise and subjective well-being

Extensive research confirms that physical exercise plays a positive role in preventing and intervening in adolescent mental health issues [8]. It not only helps alleviate psychological distress but also significantly enhances subjective well-being. As a crucial psychological indicator for adolescents, improving subjective well-being holds profound significance for meeting the people’s growing need for a better life and addressing the primary contradictions in contemporary Chinese society [7]. Research indicates that physical activity exhibits a significant positive correlation with psychological resilience and subjective well-being following traumatic events, while showing negative correlations with anxiety, depression, tension, and post-traumatic stress disorder [9]. This finding suggests that engaging in physical exercise enhances psychological resilience, alleviates negative emotions, and elevates overall life satisfaction.

Furthermore, recent research has revealed that physical exercise not only enhances adolescents’ subjective well-being but may also exert effects through multiple psychological pathways, including psychological capital, interpersonal relationships, self-esteem, cognitive reappraisal strategies, and psychological resilience [10–13]. However, some studies suggest the need to account for individual differences. Research by Netz [14] and Wicker et al. [15] indicates that physical activity may have no significant impact on subjective well-being for certain populations and may even yield negative outcomes. Thus, the relationship between physical exercise and subjective well-being remains complex and warrants further exploration across diverse groups and contexts. In summary, investigating the mechanisms through which physical exercise influences adolescents’ subjective well-being constitutes one of the core research questions of this study.

- H1: Physical exercise positively predicts subjective well-being.

### Mediating role of social support

The role of social support in individual mental health and well-being has been extensively studied, particularly as a key mediating variable between physical exercise and subjective well-being [16]. Research indicates that social support not only directly influences subjective well-being but also bridges the relationship between physical exercise and well-being [17, 18]. Social support encompasses assistance from family, friends, and other social relationships. Such support enhances psychological resilience, enabling individuals to better cope with life’s stresses and challenges [19].

Research indicates that social support mediates these relationships. Physical exercise promotes physical health and indirectly enhances well-being by expanding and strengthening social support networks. Research by Yildirim et al. indicates that social support significantly enhances life satisfaction and subjective well-being among refugee populations facing adverse environments. Particularly in adversity, robust social support systems bolster psychological resilience and promote adaptive capacity [19]. Schnittker’s research indicates that social support exerts a significant buffering effect on mental health, playing a positive role when individuals face stress and challenges, thereby substantially increasing their well-being and life satisfaction [20].

Different types of social support influence the relationship between physical exercise and subjective well-being. Lan Yiqin et al. [19] found that compared to support from friends and other sources, family support plays a more significant role in predicting an individual’s subjective well-being. This finding reveals that different types of social support contribute differently to well-being formation. Wang Feng et al. [18] validated this conclusion in medical students, demonstrating that support from family and friends significantly and positively predicted well-being. Notably, family support exhibited particularly pronounced moderating and protective effects under academic stress.

However, Li Siyue’s findings indicate that peer group and community-level social support did not significantly predict individuals’ subjective well-being [21], contrasting with previous research. This discrepancy may stem from factors such as participants’ social backgrounds, cultural differences, developmental stages, and perceived quality of support.

- H2a: Social support positively predicts subjective well-being;
- H2b: Social support mediates this relationship.

### Mediating role of psychological resilience

Physical exercise enhances subjective well-being by strengthening psychological resilience through the following mechanism: it provides individuals with controllable stress exposure scenarios (e.g., group competition, setbacks), fostering adaptive psychological resources (e.g., emotional regulation, problem-solving skills), thereby improving coping efficacy against life stressors [22]. Long-term exercisers can overcome physical challenges through physical activity, develop more positive cognitive reappraisal abilities (e.g., viewing failure as an opportunity for growth), and thereby maintain higher life satisfaction and positive emotional experiences.

Xinqiao Liu [23, 24] and others found that college students’ psychological regulation abilities can alleviate anxiety and improve health; Jiao Can’s [25] research found that psychological resilience mediates the relationship between social support and subjective well-being. Specifically: Social support provides psychological resources: Support from family and friends enhances individuals’ sense of belonging and security, reducing perceived stress, while supportive environments foster positive cognitive restructuring and improve environmental adaptability. Zhang Qiongzhi et al. argue that resilience drives well-being, with psychological resilience exerting a stronger positive effect on well-being when economic resources are abundant [26]. Individuals with high psychological resilience effectively mobilize resources to maintain emotional equilibrium and life satisfaction.

- H3a: Psychological resilience positively predicts subjective well-being;
- H3b: Psychological resilience mediates this relationship.

### Moderating role of gender

Influenced by physiological and sociocultural factors, significant gender differences exist in the relationship between social support and subjective well-being. Tao Baole et al. [27] found that men derive greater cognitive strategy enhancement benefits from physical exercise. Within the social support pathway, women demonstrate higher emotional conversion efficacy: Wang Yuze et al. [28] found that female high school students gained significantly greater psychological capital from perceiving social support than their male counterparts, and this psychological capital exerted a stronger stress-relieving effect. Yuan Yuan’s research revealed that the negative predictive effect of social support on depression was more pronounced among women, highlighting their greater proficiency in converting social support into emotional regulation resources. From a physiological perspective, men and women exhibit distinct differences that influence individual choices and coping strategies during physical exercise [29]. Under different cultural influences, men and women express emotions differently. Men typically favor confrontational and conflict-based problem-solving approaches, preferring direct resolution. Women, conversely, tend toward cooperative problem-solving, placing greater emphasis on interpersonal relationships and mutual assistance [30]. Based on the above information, the following hypotheses are proposed:

- H4a: Gender moderates the relationship between social support and subjective well-being;
- H4b: Gender moderates the relationship between physical exercise and subjective well-being.

**Fig. 1:**
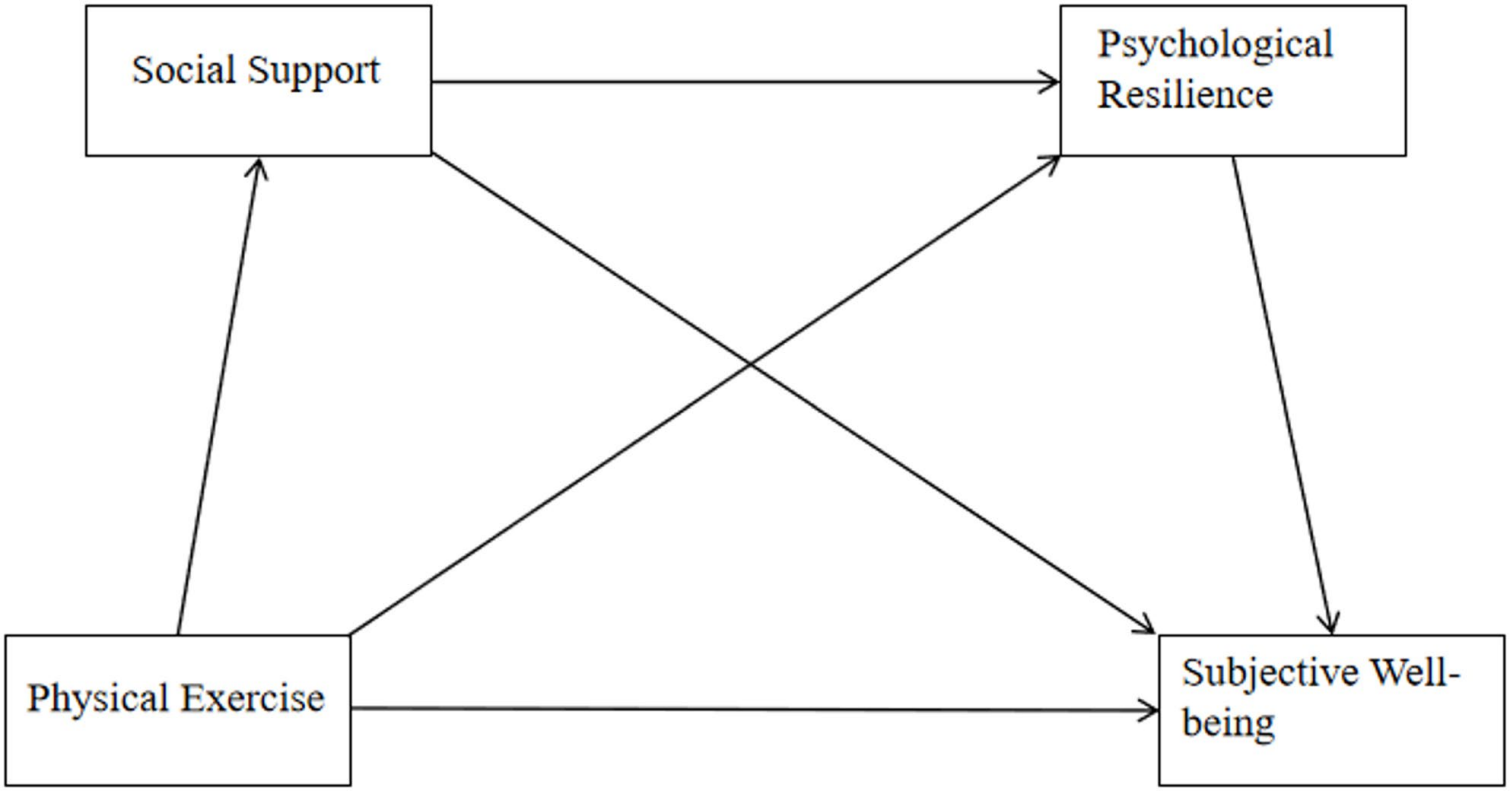
Research framework

In summary, physical exercise not only directly enhances physical health but also indirectly boosts subjective well-being through social support and psychological resilience(as shown in Fig. 1). This warrants further exploration of the mechanisms by which different types of social support and psychological resilience influence subjective well-being, as well as how intervention strategies can strengthen social support and psychological resilience to enhance subjective well-being .

## Methods

### Research participants

This study targeted university students as research participants, employing a random sampling method for questionnaire administration. Prior to questionnaire distribution, researchers provided standardized instructions to participants, clearly explaining the study’s purpose and significance. Emphasis was placed on the anonymous nature of the questionnaire to safeguard participant privacy and ensure response authenticity. A total of 564 questionnaires were distributed, with 564 returned. After quality control, invalid responses—including those completed too quickly, with missing answers, failing lie-detector tests, or exhibiting abnormal option consistency—were excluded. This yielded 494 valid questionnaires, representing an 87.59% response rate.

According to demographic data from the questionnaires, the valid sample comprised 306 males (61.9%) and 188 females (38.1%).

### Tools

#### Adolescent psychological resilience scale

The Adolescent Psychological Resilience Scale was jointly developed by Professors Hu Yueqin and Gan Yiqun in 2008. It is a standardized psychological assessment tool designed for Chinese adolescents [11, 31], aiming to evaluate individuals’ psychological adaptation and recovery capabilities when facing adversity. Comprising 27 items across five core dimensions, including goal focus, the scale comprehensively reflects adolescents’ psychological resilience levels. In this study, both reliability and validity of the scale reached statistically significant levels. The K-S nonparametric test revealed a significantly skewed data distribution (*P* < 0.05, df = 494), indicating suitability for subsequent statistical analysis. Cronbach’s α coefficients ranged from 0.71 to 0.81, demonstrating good internal consistency. The overall Cronbach’s α coefficient of 0.782 further validated the scale’s reliability within this study sample.

#### Perceived social support scale

The Perceived Social Support Scale was originally introduced by Blumenthal and developed by Zimet et al. [32, 33], later adapted for the Chinese context with appropriate revisions by scholars including Jiang Qianjin. This scale emphasizes individuals’ subjective perceptions of social support, primarily measuring their self-understanding and experienced levels of support from family, friends, and other social members. The total score reflects an individual’s overall perceived level of social support. Comprising 12 items across three dimensions—family support, friend support, and other support—the scale employs a 7-point rating scale. Characterized by operational simplicity and strong applicability, it has been widely used in assessing social support among adolescents and adults. In this study, the K-S nonparametric test yielded significant results (*P* < 0.05, df = 494), indicating reasonable data distribution. Reliability analysis revealed a Cronbach’s α coefficient of 0.862 across all three dimensions, demonstrating good internal consistency and reliability, suitable for the psychometric requirements of this study’s sample.

#### Physical activity rating scale

This study employed the Physical Activity Rating Scale (PARS-3) revised by Liang Deqing [34] to assess participants’ exercise levels. This scale evaluates an individual’s exercise volume across three dimensions, including frequency, using a Likert-type scoring method. Exercise volume is calculated to yield a final score ranging from 0 to 100, where higher scores indicate greater physical activity levels. In this study, the K-S nonparametric test results showed that the data distribution reached statistical significance (*P* < 0.05, df = 494), indicating that the measurement data possessed good distribution characteristics. Additionally, the scale demonstrated a test-retest reliability coefficient of 0.82, reflecting high stability and reliability within the study sample. This confirms its suitability for quantifying individual physical activity levels.

#### Subjective well-being scale

This study employed the Revised General Well-Being Scale [35] by Duan Jianhua to assess participants’ subjective well-being levels. This 18-item scale comprises six dimensions, including concerns about health, and employs a positive Likert-scale scoring method. In this study, the K-S nonparametric test revealed a significantly skewed data distribution (*P* < 0.05, df = 494), indicating suitability for subsequent statistical analysis. Reliability analysis revealed Cronbach’s α coefficients ranging from 0.61 to 0.68 across the six dimensions, with an overall α coefficient of 0.766. This indicates good internal consistency and sufficient reliability within the study sample, making the scale suitable for evaluating college students’ subjective well-being levels.

### Data processing

The study employed SPSS to conduct reliability and validity tests, correlation analyses, and other procedures on the primary research variables. Simultaneously, modeling analysis was performed on the four variables, specifically including tests of inter-variable correlations, chained mediation effect analysis, and moderation effect analysis. Through structural equation modeling, the path mechanisms and modes of action of each variable within the model were further explored to validate the rationality of the research hypotheses and the fit of the theoretical model.

## Research findings

### Common method bias test

To ensure the validity of research data, a common method bias test was first conducted on the survey data [36]. The results extracted 17 factors with eigenvalues greater than 1, with the first factor explaining 24.35% of the variance—below the 40% threshold.

Following the common method bias test, descriptive statistics and correlation analyses were conducted for all primary variables. Based on these results, demographic variables such as gender were incorporated as control variables in subsequent analyses. Subsequently, guided by the research hypotheses, the relationships among the four core variables—psychological resilience, social support, subjective well-being, and physical exercise—were examined. Hypotheses were tested using a moderated mediation model.

### Descriptive statistics and correlation analysis among variables

This study included 494 valid samples. Descriptive statistics and Pearson correlation analysis were conducted on four variables: Subjective Well-being, psychological resilience, Social Support and physical activity levels. The score ranges and dispersion of each variable are presented in Table 1:

**Table 1:** Mean, standard deviation, and correlation results for each variable

Variable	M	SD	Subjective Well-being	Psychological Resilience	Social Support
Subjective Well-being	80.37	13.568	—	—	—
Psychological Resilience	93.79	14.914	0.633**	—	—
Social Support	96.24	19.75	0.447**	0.707**	—
Physical Exercise	23.83	22.665	0.149**	0.171**	0.092*

*Indicates *p* < 0.05, * *indicates *p* < 0.01

The mean values of each variable are shown in the table above. Subjective well-being indicates that participants’ general happiness is at a moderately high level, with small individual differences and a distribution pattern similar to psychological resilience. Social support scored the highest, but its internal variability was greater than that of psychological resilience; psychological resilience scores were close to those of social support; exercise volume showed the greatest individual variation.

**Fig. 2:**
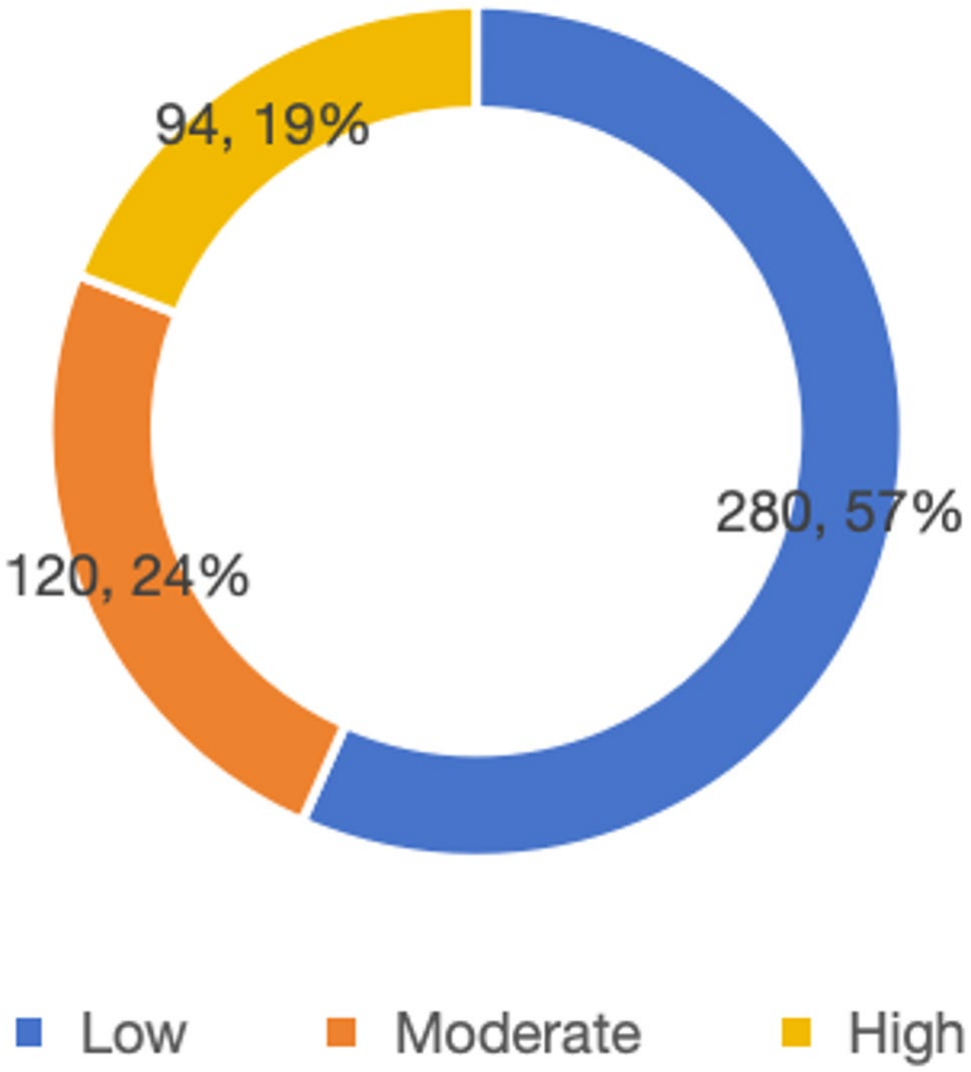
Physical fitness level chart

Based on the commonly used classification criteria of the PARS-3 scale, participants’ physical activity levels were categorized into low, moderate, and high exercise intensity groups. As shown in the figure above(Fig. 2), individuals in the low and moderate exercise intensity groups accounted for approximately 75% of the population. This indicates that the proportion of individuals in the moderate-to-high exercise intensity group is relatively small, suggesting that the overall level of physical activity participation among college students still has considerable room for improvement.

Correlation analysis revealed a positive association between psychological resilience and subjective well-being (*r* = 0.633, *p* < 0.01), confirming psychological resilience as a core determinant of well-being [37]. Psychological resilience is also positively correlated with social support (*r* = 0.707, *p* < 0.01), indicating that individuals with abundant psychological resources tend to receive greater support. The effect of exercise volume on social support is weak but significant (*r* = 0.092, *p* < 0.05), while exercise volume positively correlates with psychological resilience and subjective well-being (*r* = 0.171, *p* < 0.01; *r* = 0.149, *p* < 0.01). Increased psychological resilience, social support, and exercise volume lead to heightened subjective well-being.

### Chain mediation of social support and psychological resilience

**Fig. 3:**
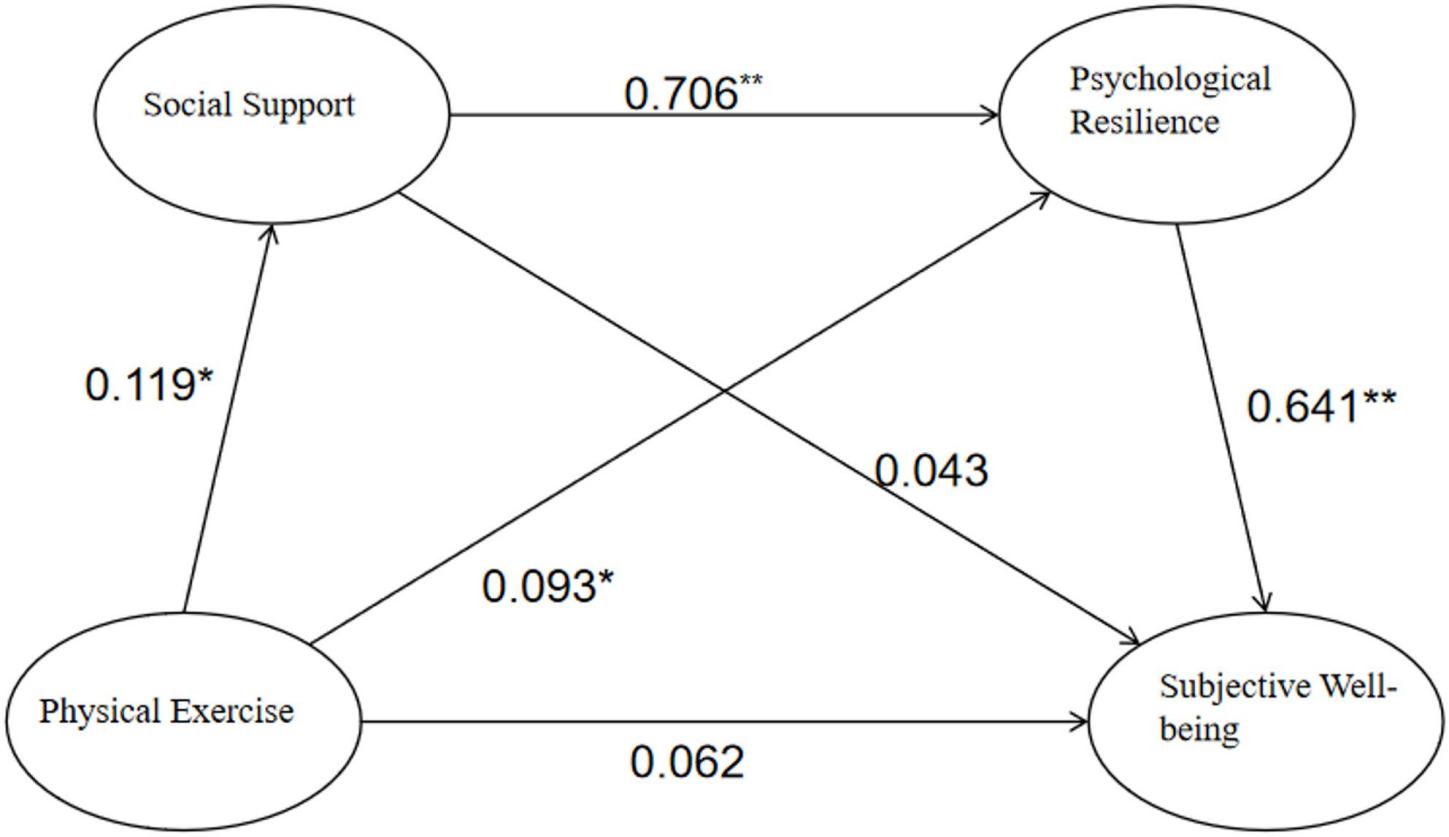
Mediation effect diagram among variables. Note: * indicates *p* < 0.05, ** indicates *p* < 0.01

The above findings indicate interrelated variables, enabling further verification of chain mediation. First, chain mediation effects were analyzed using PLS-SEM in SmartPLS [38] (as shown in Fig. 3). The figure indicates that physical exercise does not significantly predict college students’ subjective well-being (β = 0.062, *p* > 0.05). Physical exercise significantly and positively predicts an individual’s level of social support (β = 0.119, *p* < 0.05). Simultaneously, an individual’s level of social support significantly and positively predicts their level of psychological resilience (β = 0.706, *p* < 0.01). Furthermore, participation in physical exercise significantly enhances psychological resilience (β = 0.093, *p* < 0.05), and this enhanced psychological resilience directly and highly significantly promotes increased subjective well-being (β = 0.641, *p* < 0.01). However, as shown in the figure, the direct effect of social support on subjective well-being was not significant (β = 0.043, *p* > 0.05). This indicates that social support itself does not directly lead to higher well-being; its positive effect depends on enhancing an individual’s psychological resilience, which ultimately translates into increased subjective well-being [21].

This study employed Bootstrap analysis, with results presented in Table 2. As shown in the table, the effect size for the path from physical exercise to psychological resilience to subjective well-being was 0.060, with a 95% confidence interval of [0.020, 0.102], which does not include zero. This indicates a significant indirect effect. For the path from physical exercise → social support → psychological resilience → subjective well-being chain path, the effect size was 0.054, with a 95% confidence interval of [0.015, 0.095] that excluded zero, indicating statistical significance. This suggests exercise enhances well-being through a multistage transmission mechanism: first elevating social support levels, then strengthening psychological resilience, and ultimately promoting subjective well-being [39]. However, the 95% confidence interval for the path from physical exercise → social support→ subjective well-being was [-0.008, 0.020], which included zero and was non-significant. This indicates that social support alone cannot serve as a mediating variable.

**Table 2:** Bootstrap analysis for mediating effect testing

Path	Standardized effect size	95% confidence interval		Effect size(%)
		lower limit	upper limit	
Direct effect (physical exercise → subjective well-being)	0.062	-0.002	0.127	34.254
Path One	0.005	-0.008	0.020	2.762
Path Two	0.060	0.020	0.102	33.149
Path Three	0.054	0.015	0.095	29.834
Total Indirect Effect	0.119	0.063	0.177	65.746
Overall effect	0.181	0.099	0.266	

Path 1: Physical exercise → Social support → Subjective well-being; Path 2: Physical exercise → Psychological resilience → Subjective well-being; Path 3: Physical exercise → Social support → Psychological resilience → Subjective well-being

### Testing the chain moderating effect of social support and subjective well-being

**Fig. 4:**
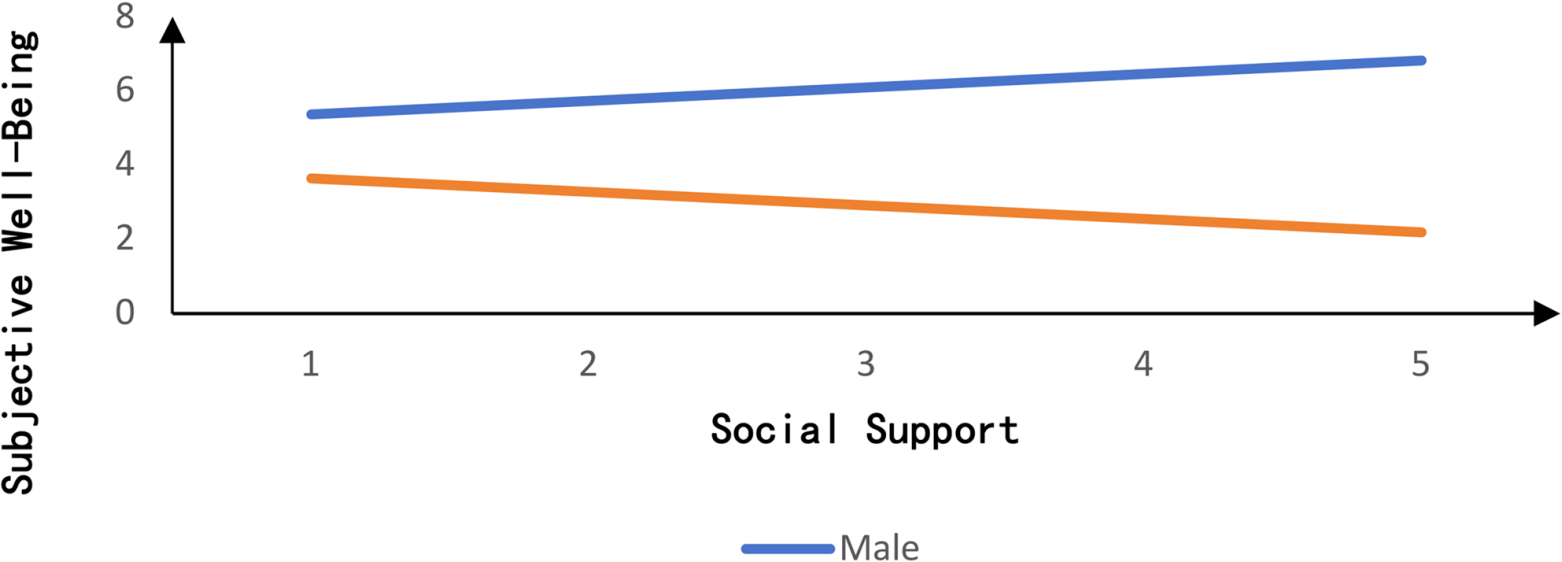
Slope diagram of gender’s moderating role in the relationship between social support and subjective well-being

Using SmartPLS, we examined the moderating role of gender in the relationship between social support and Subjective well-being. Results indicate that the interaction term between social support and gender exerts a significant negative effect on subjective well-being (β = -0.172, *p* < 0.05). To better illustrate the gender-specific moderation effect, moderation plots were generated with gender as the horizontal axis (see Fig. 4). Fig. 4 reveals that the relationship between social support and subjective well-being differs across genders [40]. At high levels of social support, gender exerts a greater influence on subjective well-being than at low levels of social support. For males, social support positively predicts subjective well-being, whereas for females, the opposite holds true: social support negatively predicts subjective well-being [41].

### Testing for moderated chain mediation effects

Moderated chain mediation effects were tested using the PLS-SEM method in SmartPLS software, with results presented in Table 3. When gender was male, the mediation effect value was − 0.043, with a 95% CI of [-0.175, 0.083] that included zero, indicating that this chain mediation effect was not significant. For females, the effect value was 0.129 with a 95% CI of [0.011, 0.242], excluding zero, indicating a significant chain mediation effect. This suggests that for females, social support plays a stronger mediating role between physical exercise and subjective well-being. The indirect effect difference was 0.172, with a 95% CI of [-0.314, -0.037], excluding zero. This indicates a significant indirect effect difference, confirming that gender significantly moderates the relationship between social support and subjective well-being.

**Table 3:** Presents the results of the moderated chained mediation effect test

Gender	effect value	Boot standard error	BootCI
Male	-0.043	0.065	[-0.175,0.083]
Female	0.129	0.060	[0.011,0.242]
Difference	-0.172	0.003	[-0.322,-0.046]

## Discussion

This study, grounded in the context of building a Healthy China and promoting college students’ mental health, delves into the mechanisms through which physical exercise influences subjective well-being among university students. It specifically examines the chain-mediated effects of psychological resilience and social support, as well as the moderating role of gender. The findings reveal the intrinsic pathways through which physical exercise enhances subjective well-being and the associated gender differences. These insights provide crucial evidence for understanding the formation mechanisms of well-being among college students and for developing targeted interventions. The study concludes as follows:

### Chain-mediated transmission mechanism of physical exercise, social support, psychological resilience, and subjective well-being

This study revealed the mediating roles of social support and psychological resilience in the research and validated the chain-mediated path of Pathway 3. The results supported the mediating roles of psychological resilience and social support in the relationship (H2b and H3b).

Contrary to the partial expectation of Hypothesis H1, this study found that physical exercise did not significantly predict subjective well-being directly. This indicates that the impact of physical exercise on college students’ well-being is not simply a matter of “exercise equals happiness” [42, 43], but its effects are primarily mediated through social support and psychological resilience [44].

Physical exercise significantly and positively predicted psychological resilience. Enhanced psychological resilience, in turn, strongly and directly promoted subjective well-being, indirectly confirming psychological resilience’s central mediating role. Bootstrap analysis further validated the significant indirect effect of “Physical Exercise → Psychological Resilience → Subjective Well-Being.” This finding corroborates the theoretical pathway whereby exercise enhances overall life satisfaction and well-being by strengthening interpersonal assistance, family support, emotional regulation, goal focus, and positive cognition in the face of adversity [45].

Research confirms that physical exercise significantly elevates social support levels, and high social support strongly predicts greater psychological resilience. Bootstrap analysis revealed a significant chained path effect: “Physical Exercise → Social Support → Psychological Resilience → Subjective Well-Being.” This indicates that engaging in physical exercise creates more opportunities for social interaction and building support networks among college students. These acquired social support resources become crucial soil for nurturing psychological resilience. Individuals with stronger resilience can then better experience and sustain well-being [13]. However, the direct effect of social support itself on subjective well-being was not significant. This clearly indicates that the role of social support in enhancing well-being must be achieved through strengthening an individual’s psychological resilience. A mere social network or perceived support, if not effectively transformed into an individual’s internal coping resources and positive mindset, is insufficient to directly enhance their subjective well-being.

### The moderating role of gender in the “social support → subjective well-being” pathway

This study further reveals that gender significantly moderates the pathway through which social support influences subjective well-being, thereby affecting the validity of the chained mediating effect.

For male college students, social support exhibits a positive predictive effect on subjective well-being, with high social support effectively enhancing men’s experience of well-being. However, the moderated chain mediation analysis indicates that among males, the chain pathway effect—“Physical Exercise → Social Support → Psychological Resilience → Subjective Well-Being”—is significant. This implies that the mechanism whereby physical exercise enhances psychological resilience through increased social support, ultimately promoting well-being, is fully realized among male college students [46].

Conversely, research indicates that for female college students, high levels of social support negatively predict subjective well-being. The moderation effect diagram reveals that under high social support, women’s well-being is significantly lower than men’s. Multiple factors may explain this phenomenon: First, societal and cultural expectations of female roles may lead women to perceive excessive attention or expectations from family and peers [47], transforming this “sweet burden” into a source of stress. Women may be more sensitive to interpersonal harmony and evaluation in relationships. For women, the “sweet burden” itself may be accompanied by higher reciprocal pressure, relationship maintenance costs, and greater concern for interpersonal harmony. These factors can translate into potential psychological stressors, thereby diminishing well-being [48]. Second, this study primarily examines individuals’ subjective perceptions of the “availability/overall level” of social support without further distinguishing its “quality,” such as whether it aligns with individual needs. Research indicates that the quantity and quality of social support exert differing effects on stress and mental health, potentially even in opposite directions [49]. Support is more likely to exert a protective effect only when it meets the recipient’s needs [50]. Some gender difference studies suggest men may be more sensitive to the quantity of support, while women are more influenced by relationship quality [51, 52]. If support lacks matching or autonomy, women may even perceive it as additional interpersonal pressure. For women, what matters is whether support is effectively transformed into internal psychological resources, not merely whether support exists.Third, adolescents may face unique body image and social anxieties during puberty, and excessive social attention (even supportive) can exacerbate these anxieties [53, 54]. Female adolescents, when confronted with frequent scrutiny from peers and social environments, are more prone to engage in appearance comparisons, self-monitoring, and negative self-evaluation concerns.

## Conclusions and practical implications

This study employed a mediation model to reflect and validate the mechanism linking physical exercise among college students with subjective well-being. First, while the direct effect of physical exercise on subjective well-being was not significant, it influenced subjective well-being through two mediating factors. Specifically: enhanced physical exercise increased social support among college students, and higher social support significantly improved their psychological resilience, thereby enabling them to perceive greater subjective well-being.However, social support itself cannot directly enhance subjective well-being. Only when it is transformed into an individual’s psychological resilience resources can it serve as a mediator between physical exercise and subjective well-being.

Second, gender exhibits a moderating effect on the path between social support and subjective well-being. This gender difference holds significant practical implications. It suggests that when designing and implementing physical exercise intervention programs aimed at enhancing well-being, gender-sensitive strategies should be adopted. For males, emphasis should be placed on fostering supportive team atmospheres and peer relationships, fully leveraging social support as an effective pathway to enhance psychological resilience and well-being. For female, greater attention should be paid to potential stressors within highly supportive environments. The focus should shift toward helping them effectively manage social expectations, enhance coping skills for interpersonal pressures, and build positive self-image and autonomy through physical activities—rather than merely increasing support density.

Schools, families, and society must fully recognize the vital role of physical exercise in promoting college students’ mental health and social development. This goes beyond mere physical fitness goals and should be integrated into the college mental health promotion system. Within physical activities, actively foster an inclusive, encouraging, and supportive team atmosphere to promote peer support and positive teacher-student interactions, thereby enhancing students’ sense of social support. Sports instruction and training should consciously incorporate elements designed to cultivate students’ abilities to overcome difficulties, cope with setbacks, manage emotions, and practice positive attribution, integrating the development of psychological resilience into the process of learning athletic skills.

While providing social support, particular attention should be given to pressures women may face. Through psychological counseling, body image education, and stress management training, they should be better equipped to navigate challenges within high-social-support environments. This approach effectively transforms social support into internal strength, avoiding one-size-fits-all happiness promotion strategies.

## Research limitations and future directions

This study also has several limitations. First, the sample data originates solely from the college student population, limiting the generalizability of the conclusions. Future research should broaden the sample scope to enhance universality while increasing sample size and striving for a more balanced gender composition. This approach will improve the robustness and applicability of the findings. Second, this study employs a cross-sectional design. Future research should adopt a longitudinal tracking design to continuously examine the dynamic relationship among physical exercise, social support, psychological resilience, and subjective well-being across multiple time points. This approach will provide stronger evidence for the temporal validity and causal direction of the chained mediation mechanism. Finally, this paper only examined the mediating and moderating mechanisms of social support and psychological resilience in the relationship between physical exercise and subjective well-being among college students. Future work could introduce additional mediating or moderating variables to further enrich the theoretical model of this relationship. Additionally, the unique phenomenon observed among female participants requires more in-depth qualitative or mixed-methods research to uncover the specific psychosocial mechanisms underlying it, thereby providing a basis for developing targeted intervention strategies.

## Supplementary Information

Supplementary Material 1.

## Data Availability

The datasets used during the current study are available from the corresponding author on reasonable request.
