# Supplementary material for: The impact of college students’ physical exercise on subjective well-being: a chain-mediation model involving psychological resilience and social support with gender as the moderator
Source: BMC Psychol. 2026 Apr 1;14:691. doi: 10.1186/s40359-026-04472-y (PMC13170145; doi:10.1186/s40359-026-04472-y)
Supplement: Supplementary file 1 — Supplementary Material 1. [file 40359_2026_4472_MOESM1_ESM.docx]

January 27, 2026

BMC Psychology

Dear Distinguished Editor,

We are pleased to submit our manuscript titled "The Impact of College Students' Physical Exercise on Subjective Well-being: A Chain-mediation Model Involving Psychological Resilience and Social Support with Gender as the Moderator" to be considered for publication in BMC Psychology as a research paper.

This study, grounded in the “Healthy China” initiative, focuses on promoting mental health among college students. Through a survey of 494 university students, it systematically examines the underlying mechanisms through which physical exercise influences subjective well-being. The study's innovations are primarily reflected in:（1）Revealing a chained mediating pathway: For the first time among Chinese university students, the complete chained mediating mechanism—“physical exercise → social support → psychological resilience → subjective well-being”—was validated, elucidating the theoretical pathway whereby social support effectively enhances well-being only by strengthening psychological resilience.（2）Identifying significant gender differences: The study not only confirmed gender's moderating role in the relationship between social support and well-being but further revealed that the aforementioned chained mediating effect was significant only among male participants, not among females. This finding challenges the common assumption that “social support necessarily enhances well-being” and offers important implications for developing gender-specific mental health interventions.

This study employed structural equation modeling (SmartPLS) for hypothesis testing, ensuring rigorous methodology and reliable results. The manuscript aligns closely with current global academic concerns regarding adolescent psychological well-being and health behavior promotion, particularly resonating with post-pandemic research demands for resilience cultivation and social connectedness. It offers clear practical significance and theoretical contributions.

We believe these findings provide new empirical evidence for understanding the mechanisms through which physical exercise promotes mental health and offer valuable insights for designing differentiated campus health promotion programs. The manuscript aligns with your journal's scope and academic standards. We hereby recommend it for your consideration.

This manuscript has not been published and is not being considered for publication elsewhere while under your review. All photos and images in the manuscript were drawn by our authors. We also confirm that all authors listed have participated actively in this study and accept responsibility for the manuscript's contents. The submission has been approved by all authors. The authors all do not have any possible conflicts of interest. Thank you very much for your consideration. Please feel free to contact me with any questions you may have. You can reach me at shenjing9264@126.com(email).

Sincerely,

Jing Shen

.JIANGSU OPEN UNIVERSITY [shenjing9264@126.com](mailto:shenjing9264@126.com)

Email: shenjing9264@126.com
